# Is Typhoid Fever Contagious

**Published:** 1896-03

**Authors:** J. W. Hargus

**Affiliations:** Carrizo Springs, Texas


### Is Typhoid Fever Contagious.

CARRIZO SPRINGS, TEXAS, January 27, 1896.

*Editors Texas Medical Journal:*

We have been having some typhoid fever, this fall and winter, in this section; however, no general epidemic, but cases here and there, for which we can not trace any adequate source. One case in one part of the country, and another twenty-five or thirty miles in an opposite direction: no other cases occurring in the same family or neighborhood, although all having the same food and drink. Not having been to any place in common, these isolated cases puzzle me to tell the source of infection.

However, I think that in one family I found the probable source. In January, 1895, I was called in consultation with Dr. F., and found three brothers, aged respectively 22, 25 and 30, down with the fever in a very bad form. The history of the cases was that a sister had been visiting in Kerr county, and had contracted a fever while there, an epidemic of which was prevailing at the time. Her parents brought her home within two weeks after getting up from the fever. The brothers, and the wife and two children, kissed the sister frequently after she came home, and were all taken with the fever within two weeks, the wife and children having gone to Colorado county on a visit meantime. The boys were past relief when I was called, and all died within a few days. The father had dosed them, so the attending physician told me, with calomel and quinine for ten or twelve days before he was called to see them; a very prevalent custom with the people in this section, which causes no end of trouble, especially when coupled with patent pills, with which the market is flooded, as well as other nostrums, which ought to be prohibited, if it were possible.

There was also another case which spread from the same source, if I am not mistaken. A young lady attending the funeral of one of the boys, kissed the sister of the boys, and contracted the fever. At least that is my opinion, as she had no other chance to contract it, not having eaten or drank anything on the premises where the sick were confined.

My reason for believing that the disease was communicated that way, and no other, is this: Of several parties that came to nurse the sick, who also ate at the place and drank the water, not one contracted the disease. And the well has been in use since that time, and no cases followed. The young lady did not eat or drink at that place, and lived ten miles from there, and was one of a family of twelve, none of which have had the fever, although drinking the water and eating the same food she had been accustomed to. My theory is that the saliva of persons will retain the typhoid fever germs several weeks after the patient is up and apparently well. You may think this far fetched, but these cases, with some others, have led me to believe that the disease may be communicated in that may. If so, why may not those isolated cases here and there, which occur in this country every fall and winter, be caused by fruit, say apples or oranges, of which considerable quantities are brought in through the fall and winter,—infected, it may be, by people lately the subjects of the fever expectorating on more or less of the fruit, or possibly by leaving the products of desquamation on the fruit? For it is a fact that all the patients do peel off like a snake, after severe fevers.

Is not this a possible source of danger that we often overlook, and may not the germs retain their vitality longer, and under conditions we do not dream of?

Now, before we had railroads, and easy and quick means of transportation to this portion of the country (that is, from 1876

to 1880), we had very few cases of typhoid fever. But since that time, we have plenty of it (in the fall and winter).

So far as the sources of water supply are concerned, in this county they are varied: some receiving it from living springs, some from dug wells, some from artesian wells, and some from creeks, lakes, and ponds. And so far as my observations go, one class has about as much typhoid fever as the other, rich and poor alike.

I would lkie to hear from some of the brethren about the matter. Success to the little "Red-Back."

Yours truly, J. W. HARGUS, M. D.

